# Risk Prediction, Diagnosis and Management of a Breast Cancer Patient with Treatment-Related Cardiovascular Toxicity: An Essential Overview

**DOI:** 10.3390/cancers16101845

**Published:** 2024-05-11

**Authors:** Michael Cronin, Aoife Lowery, Michael Kerin, William Wijns, Osama Soliman

**Affiliations:** 1 School of Medicine, University of Galway, H91 V4AY Galway, Ireland; 2 Precision Cardio-Oncology Research Enterprise (P-CORE), H91 TK33 Galway, Ireland; 3 CURAM Centre for Medical Devices, H91 TK33 Galway, Ireland; 4 Discipline of Surgery, Lambe Institute for Translational Research, University of Galway, H91 V4AY Galway, Ireland

**Keywords:** cardiology, risk prediction, breast cancer, cardiac CT, echo, cardiac MRI, nuclear medicine, heart failure

## Abstract

**Simple Summary:**

This is a contemporary overview of how cardiology issues arise in the breast cancer population from anti-cancer agents (e.g., chemotherapy, targeted therapy, endocrine therapy, and radiotherapy). The overview demonstrates how we identify these cardiac issues and what we can do about them.

**Abstract:**

Breast cancer is amongst the most common invasive cancers in adults. There are established relationships between anti-cancer treatments for breast cancer and cardiovascular side effects. In recent years, novel anti-cancer treatments have been established, as well as the availability of multi-modal cardiac imaging and the sophistication of treatment for cardiac disease. This review provides an in-depth overview regarding the interface of breast cancer and cancer therapy-related cardiovascular toxicity. Specifically, it reviews the pathophysiology of breast cancer, the method of action in therapy-related cardiovascular toxicity from anti-cancer treatment, the use of echocardiography, cardiac CT, MRI, or nuclear medicine as diagnostics, and the current evidence-based treatments available. It is intended to be an all-encompassing review for clinicians caring for patients in this situation.

## 1. Introduction

Cardio-oncology is a relatively new area of interest and trial data are limited, with expert consensus often used in current guideline recommendations [1]. Patients are surviving longer with cancer due to advances in screening and treatment [2], which has led to an increase in the prevalence of downstream complications. As such, the recent area of cardio-oncology has gained a foothold as a potential area for patient improvement [3]. Key tenets of this review are cancer therapy-related cardiovascular toxicity, and cancer therapy-related cardiac dysfunction.

Dedicated services in the United Kingdom have found a 10-fold increase in cardio-oncology referrals to a dedicated clinic in the Royal Brompton Hospital between 2016 and 2021 [4]. The majority of patients referred to this clinic had a diagnosis of breast cancer. With their supervision, the vast majority of patients who experience cancer therapy-related cardiovascular toxicity then proceed to safely complete their cancer treatment.

This review aims to explore the background to this topic, what diagnostics are available to diagnose cancer treatment-related cardiovascular toxicity, and the therapeutics available to patients.

## 2. Overview of Cancer Therapy-Related Cardiovascular Toxicity

A core concept in the realm of cardio-oncology is that of cancer therapy-related cardiovascular toxicity (CTR-CVT) [5], and the role that a cardio-oncologist plays in its prevention [1]. Cancer therapy-related cardiac dysfunction (CTRCD) is a separate, recommended term to describe cardiomyopathy, an issue specific to the muscle of the heart. Due to its large prevalence, this area has been assigned its own criteria. See the below table (Table 1) for an overview of CTR-CVT and CTRCD (adapted from European Society of Cardiology (ESC) guidelines), with the commonly encountered causative agents.

Typical causative anti-cancer treatments for breast cancer will be discussed subsequently. These agents used in breast cancer will not encompass all the anti-cancer agents that are listed in the below table. To maintain a consistent narrative the review will focus on those agents (and CTR-CVT) that are common and relevant in breast cancer patients. This review is not intending to provide an overview of all possible anti-cancer treatment cardiovascular toxicities.

## 3. Breast Cancer Histology and Grading

At a preclinical level, consensus has led to five major intrinsic breast cancer subtypes: Luminal A, Luminal B, HER2-enriched, basal-like, and claudin-low-type [6,7]. At a clinical level, breast cancers are classified by their hormonal status. Luminal breast cancers are the most common type in Western populations (c 70% of all breast cancers) [8], and usually are an invasive breast cancer of no specific subtype (but may also differentiate into tubular, lobular, mucinous, amongst others). They are both oestrogen receptor-positive, and separated into Luminal A and B by proliferation-related and luminal-related pathways, with Luminal B having the worse prognosis [9]. HER2-enriched cancers express a high level of HER2 and are negative for oestrogen and progesterone receptors [9] with a poor prognosis. It typically makes up 10–15% of encountered breast cancers. Basal-like cancers (otherwise known as triple-negative breast cancer) occur more commonly in young women and in African-American populations, do not express any oestrogen, progesterone, or HER2 receptors, and are associated with a poor prognosis [10]. Typically, these make up 20% of all breast cancers. Claudin-low tumour types are typically receptor-negative and again carry a poor prognosis [11].

At baseline, diagnosis and prognosis are established via both the American Joint Committee on Cancer [12]—a standardised approach of tumour size (T), nodal status (N), and metastases (M)—with biological factors (HER2, progesterone, and oestrogen status), and grading. Grading is undertaken by the Elston–Ellis modification of the Scarff–Bloom–Richardson system [13]. Expression of the oestrogen receptor is undertaken to determine those that would benefit from endocrine therapy—oestrogen receptor downregulators or modulators, and third-generation aromatase inhibitors [14]. Expression of the progesterone receptor is predominantly a positive predictive sign in terms of overall survival, and time to recurrence/treatment failure [15]. Inversely, the lack of progesterone expression is considered a negative prognostic sign. HER2 expression is considered an aggressive finding, with higher rates of disease recurrence and metastases [16]. Ultimately, with all this information, a patient in a clinical setting may, as an example, be described as T2N1M0 G3 ER + PR + HER-.

## 4. Anti-Cancer Treatment

In general, treatment strategies are guided by the TNM system, tumour location/grade, and clinical factors such as burden of disease and performance status. Early breast cancer can be treated with either breast-conserving surgery or mastectomy [17], which can include axillary lymph node clearance. In general, there is no reason to distinguish this subset of patients from the general population when considering cardiac disease.

Systemic anti-cancer therapy (SACT) can be neoadjuvant (pre-surgery) or adjuvant (not pre-surgery), and this is individualised to the patient after diagnosis. It can be used for large local tumours, nodal involvement, or metastatic disease. The current agents that are predominantly used [9] are listed in the following table (Table 2), and subsequently elaborated upon. Radiotherapy can be applied to breast tissue or the chest wall as the clinical situation allows for, and will improve survival and rates of recurrence [18]. It is further discussed below.

Endocrine therapy has a particular indication for oestrogen- and/or progesterone-expressive breast cancers, improving cancer-related mortality [19]. Examples of agents include modulators (Tamoxifen), downregulators (Fulvestrant), and aromatase inhibitors (Anastrazole). Given that oestrogen is expressed in 70–75% of invasive breast cancers [20], these are very commonly used agents.

Targeted therapy is used for HER2 breast cancers and includes therapies such as trastuzumab, pertuzumab, and lapatinib. A significant advance was achieved in recent times in the form of antibody–drug conjugates [21]. Trastuzumab–deruxtecan (a topoisomerase 1 inhibitor) is one such example, gaining a tissue-agnostic accelerated approval by the Food and Drug Administration in the United States of America [22].

The following tables (Table 3, Table 4, Table 5 and Table 6) are simplified from the European Society of Medical Oncology [23,24] to help understand the guidance of which anti-cancer treatments to use for different situations.

### 4.1. Systemic Anti-Cancer Treatment

#### 4.1.1. Carboplatin

There are few reports regarding a cardiac dysfunction profile of carboplatin, with myelosuppression the major clinical side effect encountered [25]. Cardiac side effects, although rare, include myocardial infarction, vasospasm, and venous/arterial thrombotic events.

#### 4.1.2. Cyclophosphamide

Often used as a combination therapy for breast cancer [26], cardiac toxicity is rare and associated with high doses and combination with other chemotherapy (e.g., anthracyclines, cisplatin) or concomitant radiotherapy involving the mediastinum. The mechanisms of toxicity are felt to be myocyte and endothelial injury via destructive metabolites [27], and clinically the patient would most commonly present with heart failure syndrome or with atrial fibrillation.

#### 4.1.3. Fluoropyrimidines

The method of cardiovascular toxicity from these agents is felt to be multifactorial: oxidative stress with cell damage, diminished ability to transfer oxygen by red blood cells coupled with an increase in metabolism causing ischaemia, and endothelial damage/thrombosis [28]. The incidence is as high as 10% [29], and the clinical picture is of chest pain with acute ECG changes due to coronary vasospasm and/or myocardial infarction [30].

#### 4.1.4. Taxanes

The absolute risk of cardiac disease from taxanes is unknown, but is felt to be minimal. The literature regarding the relationship is indeterminate; however, a previous report suggested it is a safer option than anthracyclines in patients with pre-existing systolic dysfunction. [31]. This is at odds with a prospective study regarding paclitaxel where 20% (N = 50) suffered cardiotoxicity by ejection fraction at 30 months [32].

#### 4.1.5. Anthracyclines

The relationship is well described in this anti-cancer treatment [33] and the cardiac toxicity is via the creation of reactive oxygen species [34]. These molecular changes clinically manifest as a heart failure syndrome, which, in the vast majority of patients, occurs early (<1 year since treatment commencement) and is an asymptomatic process [35]. The patients who tend to experience anthracycline-induced cardiac effects are >65, female, receive concomitant radiotherapy to the mediastinum, have pre-existing cardiac disease, have kidney failure, undergo concomitant alkylating/immunological chemotherapy, and total a cumulative lifetime dose of >400 mg/m^2^ [2].

#### 4.1.6. Immune Checkpoint Inhibitors

These agents block inhibitory molecules on T cells (namely, CTLA4 and PD1), and antigen-presenting cells (PDL1) to induce an antitumour response [36]. However, the deficiency of these pathways weakens the overall immune system of myocardial cells, making them vulnerable to cardiovascular side effects. Cardiac adverse events are quoted around 0.3–1.9% [37], with myocarditis, pericarditis, systolic dysfunction, arrhythmia, and myocardial infarction listed within these events. Myocarditis is a severe clinical entity when it arises in this population, with a mortality of 50% [38].

#### 4.1.7. Cyclin-Dependent Kinase 4/6 Inhibitors

These agents are used in oestrogen-positive/HER2-negative metastatic breast cancer patients, and they have a relationship with prolongation of the corrected QT interval on ECG [39].

#### 4.1.8. HER2-Specific Tyrosine Kinase Inhibitor

Tucatinib is indicated for active breast metastases from a breast cancer primary in conjunction with trastuzumab and capecitabine. In the clinical trial [40] regarding its use, 4% of patients (overall population N = 50) experienced grade 1 heart failure. Lapatinib, another example, has additional action at the epidermal growth factor receptor.

#### 4.1.9. Nucleoside Analogues

Gemcitabine is indicated for use in triple-negative breast cancer that is positive for programmed-death ligand 1, where it is combined with carboplatin and pembrolizumab. Although uncommon, cardiovascular side effects include myocardial infarction, heart failure, supraventricular arrhythmias, and pericardial effusions secondary to capillary leak syndrome [41].

### 4.2. Radiotherapy

Radiotherapy can lead to valvular, coronary, myocardial, and conduction disease [42], and is more prevalent in the left breast due to proximity to the heart [43]. Valve disease has a latent period of 10–20 years on average [44], with incidence increasing when the cardiac absorbed dose exceeds 30 Gray. Coronary disease in breast cancer patients treated with radiotherapy tends to predominate in the left anterior descending artery [45], again postulated secondary to anatomical placement of the coronary vessel in comparison to the left circumflex and right coronary arteries. Conduction disease varies in significance; however, there is a described relationship between mediastinal irradiation and advanced atrio-ventricular conduction disturbances [46], as there is between breast cancer radiotherapy and various pericardial diseases [47].

### 4.3. Endocrine Therapy

There is a heterogeneous group of effects from this cohort of therapies. Tamoxifen (modulator therapy) reduces the risk of myocardial infarction, ischaemic heart disease, and heart disease-related death, and has favourable effects on lipid profile [48], but at the population level is associated with increased risk of stroke and venous thromboembolism. This generally favourable effect on risk profile was more significant in modulator therapy than aromatase inhibitor therapy [49]. Aromatase inhibitor therapy does not have a direct correlation with an increased risk of myocardial infarction, but given that it increases patient life expectancy, patients on this therapy are more likely to experience non-cancer-related death as they grow older [50]. As opposed to Tamoxifen, Exemestane (an aromatase inhibitor) is less likely to cause venous thrombo-embolic disease [51]. Overall, aromatase inhibitors are better tolerated by patients in comparison to tamoxifen; however, tamoxifen carries a slightly improved cardiovascular risk profile, and endocrine therapy risks are indirect, with an increase in cardiovascular risk due to the lengthening of a patient’s life span [52].

### 4.4. Targeted Therapy

Targeted agents against HER2 (e.g., trastuzumab, pertuzumab) induce protein and mitochondrial change, but not cell apoptosis, which causes the cardiotoxicity side effects that it is associated with to remain a reversible process [53]. A total of 10% of patients have their treatment ceased due to cardiac side effects (predominantly left ventricular systolic dysfunction), with those at most risk > 60 years of age, non-Caucasian, baseline arterial hypertension, BMI > 25, and baseline LVEF < 60% [54]. A patient clinically presents with a heart failure syndrome, as described in the initial Section describing definitions of cancer therapy-related cardiac dysfunction.

## 5. Cardiac Biomarkers

Cardiac biomarkers represent an important component within the care of cardio-oncology patients, and are included in the ESC guidelines in 2022 for baseline assessment, recognition of CTRCT, and monitoring of recovery [1]. The vast majority of the literature refers to their use in HER2 and anthracycline therapy. They do not, by themselves, indicate cessation of an anti-cancer treatment. However, when elevated they may prompt more intensive monitoring or further investigations. When defining an elevation in cardiac biomarkers, previous literature has offered “any value above the laboratories normal reference range” as the definition [55].

Previous meta-analysis (10 studies, 462 patients) [56] found those with an elevated troponin on HER2 and/or anthracycline treatment carried a higher risk of left ventricular systolic dysfunction. This analysis did not find that the presence of natriuretic peptides was consistently associated with a decrease in LVEF. Some of the individual studies from this meta-analysis demonstrate the difficulty suggested by the ESC guidelines in establishing a homogenous consensus towards cardiac biomarkers. One study looked at trastuzumab-induced cardiotoxicity at 6 and 9 months in patients who also received anthracyclines (61 total, 18 affected patients), and did not find an association between NT-pro-BNP and CTRCD [57]. In direct comparison, a study of 26 breast cancer patients compared with an age- and sex-matched healthy control group, found brain-natriuretic peptide to be an independent predictor of LVEF, when significant LVEF reduction was taken to be >10% [58]. Similar positive predictive findings regarding natriuretic peptides (NT) were published by Urun et al. [59] in a population treated by monotherapy trastuzumab, or combination therapy, over a median period of 14.5 months of observation. Blancas et al. [60] found natriuretic peptides above the upper limit of normal, when adjusted for age and diabetes, to potentially be associated with a higher risk of trastuzumab-related cardiotoxicity during treatment (N = 66). A total of 27 patients suffered cardiotoxicity, with 66.7% of those labelled with cardiotoxicity found via cardiac symptomatology. Of note, >90% of patients received radiotherapy, and one/both of anthracyclines and taxanes.

Another study looked at 251 patients treated with trastuzumab for breast cancer, with 17% suffering cardiotoxicity (N = 41) [61]. These authors found Troponin-I elevation (N = 36) either at baseline or during trastuzumab treatment to be an independent predictor of trastuzumab-induced cardiotoxicity. Kitayama et a. [62] published 40 patients (N = 18/45% on trastuzumab) with breast cancer treated with trastuzumab. Their analysis suggested that any rise in high-sensitivity Troponin T was associated with cardiotoxicity (LVEF drop > 10% in their study). Interestingly, the absolute value of the troponin rise had no association with a cardiotoxic outcome. Lastly, Sendur et al. [63] reviewed 164 patients (N = 108 with 9-week treatment, N = 56 with 52-week treatment) on adjuvant monotherapy trastuzumab for HER2 breast cancer. At 32-month follow-up, they found no significant difference in biomarker levels between the two groups but found a correlation with raised CRP and NP in their cardiotoxicity cohort. Bell et al. [64] published a cell viability study via Western blot analysis and polymerase chain reaction of cardiomyocytes in patients treated with trastuzumab and epirubicin. In those in the combined treatment arm, cell viability decreased, as well as a significant increase in troponin-I and BNP levels, suggesting a positive predictive value of the biomarkers.

Alternatively, other researchers that are cited in the literature [65,66,67,68,69,70] have found no predictable association between cardiac biomarkers and trastuzumab-induced cardiotoxicity. The absolute numbers within these populations were small, with 15, 3, 24, 11, 10, and 5 patients overall reaching the cardiotoxicity definition, respectively, within these studies. Of note, the NeoALLTO sub-study [71] reviewed a population treated with either trastuzumab or lapatinib, or a combination. In total, 39 patients in this trial (13.9%) had an elevated NT-proBNP, with only 1 experiencing a cardiac event. Indeed, these authors expressed doubts regarding the clinical relevance of biomarker trending. Zardaras et al. [72] concluded in a population of 533 (N = 2 achieving a primary cardiac endpoint and N = 31 achieving a secondary cardiac endpoint) that cardiac troponin and NT did not carry a predictive value in achieving a cardiac endpoint or significant LVEF drop. Similar conclusions were found by Posch. et al. [73] (N = 185, 19/185 experiencing cardiotoxicity) and Canale et al. [74]. Immune checkpoint inhibitor therapy is associated with myocarditis, and the elevation of Troponin T correlated with the severity of clinical presentation [75]. Conversely, in those with deteriorating cardiac function treated with cyclophosphamide, natriuretic peptides were elevated to a greater extent than in those who had normal cardiac function [76].

Frequently, trastuzumab is coupled with anthracycline-type chemotherapy, and multiple studies have looked at this population with a view towards biomarkers. Putt. et al. [77] found that patients treated with adjuvant anthracycline therapy, followed by taxane and trastuzumab therapy, had a significant elevation in some biomarkers after 3 months of treatment. This study included 78 patients, with 23 experiencing 39 events by 15 months. High-sensitivity cardiac troponin-I was one of the biomarkers that saw a rise, NT-pro-BNP was not. Of note, this associated rise in troponin was not associated with the group experiencing cardiotoxicity. Papa et al. [78] followed 179 patients, at low cardiovascular risk, over 5 years, with non-metastatic breast cancer treated with anthracycline ± trastuzumab. In total, 53 (30%) developed an elevated cardiac troponin I, with mean LVEF 60 ± 6.5% at 1 year, compared with 62 ± 4.2% when troponin I remained below the upper limit of normal. They concluded that troponin I correlated with their early alterations in LV systolic function, but did result in major adverse cardiac events. The issue with these populations, as described previously by Triggiani et al. [79], is the difficulty of separating cardiac events introduced by anthracyclines vs. trastuzumab.

Bonsignore et al. [80] measured VO2 peak in 147 patients at 6 ± 2 weeks after completion of trastuzumab and did not establish a correlation between natriuretic peptides, troponin, and V02 peak. Global longitudinal strain, E:e’ measurement on tissue Doppler imaging, and age per ten years were found to be related to V02 max. This points to a key trend within biomarkers for cancer therapy-related cardiotoxicity, in that in current practice, their main use is as a clinical adjunct. Beyond echocardiographical imaging for toxicity detection, cardiac MRI also has established clinical indications. Altaha et al. [81] did not detect a relationship between troponin, natriuretic peptides, and T1 or T2 mapping sequences in those with, or without, cardiotoxicity. Indeed, this study included 10 HER2 patients with cardiotoxicity, 10 without, and 30 healthy subjects. A separate publication had suggested similar findings previously [82], where ultra-sensitive troponin-I was an independent predictor of cardiotoxicity amongst 78 patients with breast cancer who underwent both doxorubicin and trastuzumab therapy, but not with LVEF reduction. Sawaya et al. [83] presented 81 patients with HER2-positive breast cancer who received anthracycline, taxane, and trastuzumab therapy. A total of 32% (N = 26) developed CTRCT, with 5/26 symptomatic. Ultra-sensitive troponin I (as well as peak systolic myocardial longitudinal strain assessment), when measured at the completion of the anthracycline course (most commonly 3 months after last anthracycline dose), predicted subsequent cardiotoxicity. This carries clinical significance as it will help guide the clinician responsible for therapeutic adjustments, closeness of monitoring, and introduction of cardioprotective medications.

Other biomarkers were myeloperoxidase (MPO), placental growth factor (PIGF), and growth/differentiation factor-15 (GDF-15). These biomarkers are mentioned in separate literature as a future direction for biomarker monitoring [84,85]. MPO is created by polymorphonuclear leucocytes [86], causing an inflammatory reaction via oxidative stress. This reaction in an extreme fashion has a hypothesised relationship with doxorubicin, but not with trastuzumab [87]. PIGF shows promise, as Putt. et al. [77] suggest a cardioprotective role through angiogenesis promotion, given previous suggestions that trastuzumab affects angiogenesis. Insulin-like growth factor 1 (IGF-1) has been shown to decrease concomitantly with inflammation and oedema on cardiac MRI during trastuzumab therapy [88], with both demonstrating a relationship with increased cardiovascular events in a general population.

Cardiac Myosin Lightchain 1 (cMLC-1) is presented by Yu et al. as a novel biomarker [89]. cMLC-1, playing an important role in cardiac muscle contractility, was measured in 79 breast cancer patients between 2018 and 2020 (HER2 + ve = 40, HER2 − ve = 39). In 4 patients, elevated cMLC-1 was found, with 3 of these patients developing trastuzumab-induced cardiomyopathy. Largely hampered by its low numbers, this study did provide a basis for a new type of biomarker moving forward.

Separately, interleukin-33R, otherwise known as suppression of tumorigenicity 2 protein/interleukin-33R (ST2/IL33-R), has been assessed in small numbers as another novel biomarker in 22 patients who receive trastuzumab, pertuzumab, and docetaxel for HER2 breast cancer [90]. When followed up by echocardiography at 6 months post-treatment, an average of a 4% LVEF reduction was found. NT-pro-BNP had a statistically significant correlation with LVEF reduction in this population. ST2/IL33-R readings in isolation were stated to be statistically significant.

Thompson et al. [91] published original data regarding Paraoxonase-1 (PON-1) activity in a breast cancer population (N = 184) treated with doxorubicin ± trastuzumab (17.8% received), including both HER2-negative and -positive patients. A decrease in LVEF < 50% or ≥10% from baseline happened in N = 22 (12%) of the doxorubicin group, and in N = 12 (33.3%) of the combination therapy group. Each increase in PON-1 by 10% was associated with an increase in cancer therapy-related cardiotoxicity in the doxorubicin group. The authors concluded that PON-1 had a promising role in cardiotoxicity, but it remains to be seen how much significance it carries when distilled to patients on trastuzumab.

Interleukin 6 (IL6), a pro-inflammatory cytokine coupled with an anti-inflammatory myokine, was assessed within a biomarker panel and published in 2018 by Yu et al. [92]. In their study, cardiotoxicity was defined as the development of NYHA 3–4 symptoms, or LVEF decrease of >10% to <53%, or >16% total decline. Interleukin 6 elevation at baseline was found to have an association with the 11/80 patients who prospectively developed cardiotoxicity. Some clinical preclinical analyses of foetal cardiomyocytes exposed to trastuzumab found a reduction in IL6 along with an increase in cell viability when treated with dapaglifozin, a sodium-glucose cotransporter 2 inhibitor, and berberine, a nutraceutical compound [93,94]. This, when taken into consideration with the previously mentioned cardiomyocyte viability testing [64] in those on trastuzumab treatment, suggests a potential future benefit in depressed ejection fraction secondary to cancer treatment. However, IL6 monitoring is not currently in the ESC cardio-oncology guidelines for trastuzumab monitoring [1], and Mantovani et al. [95] echoed this sentiment when early myocardial dysfunction found via “speckle tracking” on echo did not correlate with an elevation in IL6 for their HER2 population. This study also included tumour necrosis factor-alpha (TNF-a) as a measured biomarker, with again no correlation found.

Circulating cell-free DNA (cfDNA) determined to be of cardiomyocyte origin by methylation was reviewed by Moore et al. [96] in their clinical trial published in 2022. They analysed samples from 29 patients who received doxorubicin and cyclophosphamide at least 2 months prior, and then every 3 months during trastuzumab therapy. N = 5 developed cardiotoxicity (NYHA 3 or 4 heart failure, or EF decline ≥ 10% to below 53% or ≥16%), and the maximum level of cfDNA was elevated in those who experienced cardiotoxicity. A cut-off of >10 copies of cfDNA was felt to be a promising biomarker, albeit at a preliminary stage.

A genomic analysis was published by Nakano et al. [97], where 11 patients had suffered cardiotoxicity secondary to trastuzumab. A genome-wide association study was performed and compared to a population of 257 controls. Five loci were postulated to have a genetic contribution: rs9316695 on chromosome 13q14.3, rs28415722 on chromosome 15q26.3, rs7406710 on chromosome 17q25.3, rs11932853 on chromosome 4q25, and rs8032978 on chromosome 15q26.3. Alternatively, the Cardiotoxicity of Adjuvant Trastuzumab Study (CATS) presented by Goel et al. [98] assessed germline single-nucleotide polymorphisms as part of their analysis of 222 patients across 17 centres. These patients received anthracyclines followed by 12 months of trastuzumab. Cardiotoxicity was defined as “cardiac death, NYHA class 3/4 heart failure, grade 3/4 arrhythmia/ischemia, drop in LVEF > 15% from baseline, or drop in LVEF of >10% to <55%”. ERBB2, FCGR2A, and FCGR3A were the single-nucleotide polymorphisms analysed. In their study, no predictive ability in their genomic analysis was found. Although with mixed early reviews, especially given a lack of heretofore dependable serum biomarkers, genomic analysis may have a role in the care of breast cancer patients.

## 6. Echocardiography

Echocardiography is the bedrock of clinical cardiology. It is a cheap, safe, and readily available test that yields essential diagnosis and prognostic information. The following is an overview of its use framed by the context of this review, and is by no means all-encompassing of its clinical utility.

### 6.1. The Standard Examination

The following examination is adapted from the American Society of Echocardiography Guidelines for performance of transthoracic echocardiography in adults [99]. For any patient at screening prior to cancer treatment, and for diagnosis of treatment-induced cardiac toxicity, this table (Table 7) includes the essential elements with some optional features.

### 6.2. Cardiomyopathies

There are multiple more cardiomyopathies than this review has scope to cover (e.g., hypertrophic, arrhythmogenic, storage-related, non-compaction) and, thus, only the most prevalent cardiomyopathies to breast cancer patients who experience cardiotoxic side effects are included. Many of the anti-breast cancer agents listed above (anthracycline, HER2 agents, cyclophosphamide, Immune Checkpoint Inhibitors) are associated with ventricular systolic failure, and clinically this is the most common clinically encountered cardio-oncology referral [4]. The following paragraphs will describe how this is discovered through echocardiography.

#### 6.2.1. Left Ventricular Systolic Function

The most common clinically significant cardiomyopathy in this review is the left ventricle. Atrial myopathies and right ventricular myopathies are important clinical entities; however, in this patient population, they are often secondary findings. The left ventricle is assessed by internal linear dimensions, volumetric measures (using 2D or 3D), and the ejection fraction (typically by Simpson’s biplane method of discs) [100]. The left ventricle’s systolic and diastolic function, as well as wall thickness, should be indexed with the patient’s body surface area and gender. An LV ejection fraction is simply calculated ((LV end-diastolic volume—end-systolic volume/LV end-diastolic volume) × 100), with a normal systolic function being 63 ± 5%.

Strain is considered an equivalent of function, and can be observed in the longitudinal, radial, or circumferential directions, with global longitudinal strain the most commonly accepted method of left ventricular deformation assessment. Normal values of global longitudinal strain range from −15.9 ± 6%. It is acquired via speckle tracking, where echo-bright features within the ultrasound picture (i.e., speckles) are followed from frame to frame. When given a period of time, the displacement rate can be used to calculate velocity, motion, strain, and strain rate. An ejection fraction that remains in the normal range for a patient cannot with certainty rule out myocardial dysfunction, with deformation imaging emerging as an improved prognostic tool in comparison [101].

#### 6.2.2. Left Ventricular Diastolic Function

Diastole is the heart in the relaxation part of the cardiac cycle. Diastolic dysfunction is a separate entity, which is often experienced in a heart failure syndrome where the ejection fraction is preserved. In echocardiography, this is observed through the left atrial size, the mitral inflow signal (for E and A wave, and deceleration time), pulmonary venous flow, and tissue Doppler imaging (for e’ measurement). Diastolic heart failure is a common clinical entity in the general population, which shares common clinical features with those who experience cancer. After hospitalisation for heart failure with preserved ejection fraction, the 5-year survival is 35% [102]. Of the anti-cancer treatments discussed in this review, anthracycline chemotherapy and radiotherapy have established relationships with heart failure and preserved ejection fraction. Left atrial volume is often established during this portion of the assessment, which is relevant to cyclophosphamide-induced atrial fibrillation. Patients with enlarged left atrial volumes are less likely to maintain sinus rhythm.

#### 6.2.3. Right Ventricular Function

RV function is more complex to estimate via echocardiography secondary to its non-elliptical shape, method of contraction, and dependence on loading conditions. Generally, in present practice, it comprises chamber size, longitudinal motion (the tricuspid annular plane motion), fractional area change, septal eccentricity index, and the s’ (tissue Doppler imaging through the lateral RV annulus). The systolic pressure of the RV can be calculated in mmHg from a tricuspid regurgitation jet using the equation (TR Max Velocity × 4). A patient suffering cancer therapy-related cardiac dysfunction may have dysfunction of the right ventricle, which, if present, is most likely experienced via symptomatic CTRCD, as it is not necessary to diagnose asymptomatic CTRCD. Right ventricular failure symptoms include pedal oedema, elevation of the jugular venous pressure, and abdominal ascites.

### 6.3. Ischaemic Heart Disease

Echocardiography is recommended as a nearby assessment tool for acute chest pain [103]. It is not the ultimate test in terms of ischaemic heart disease, but it carries a role in the identification of regional wall motion abnormalities using the 17-segment model (particularly in instances of diagnostic uncertainty), in the identification of mechanical complications of myocardial infarction (ventricular septal defect, mitral regurgitation, LV thrombus), and in the role in/out of alternative diagnoses to acute ischaemic heart disease (e.g., acute aortic disease, pulmonary embolus). Lastly, it carries a prognostic ability in the evaluation of the LV ejection fraction, which, when lowered, is associated with a poorer prognosis. The relevant population in this instance would include those breast cancer patients who have undergone previous radiotherapy, or are on fluoropyrimidine therapy or endocrine therapy, as discussed in the subsection regarding anti-cancer treatment.

### 6.4. Valvular Heart Disease

Again, a broad topic; the method of different valvular acquisition methods is listed in the subsection above on the standard examination. The role of this review is not to provide an expert opinion on how each possible valvular heart disease is diagnosed, but rather to flag the relevant disease processes of breast cancer-induced cardiovascular toxicities and discuss them as an overarching narrative during this review. Valvular disease in this population has a higher incidence in those with previous radiotherapy exposure, which typically leads to stenotic lesions. Ventricular systolic/diastolic failure from systemic chemotherapy/targeted therapies can lead to secondary mitral regurgitation, the specific features of which are in the below subsection on transoesophageal echocardiography. Despite these findings, societal guidelines consider no difference in the evaluation of cancer patients vs. the general population. Valvular heart disease employs a multi-modal approach, which is touched upon below in the use of cardiac CT for valvular heart disease.

### 6.5. Pericardial Disease

Echocardiography is the first-line examination in suspected pericardial disease diagnosis. It can diagnose and quantify a pericardial effusion, assess for haemodynamic consequences/tamponade, and assess for pericardial constriction. These echocardiographic findings include diastolic collapse of the right ventricular free wall, swinging motion of the heart in the pericardial sac, a dilated IVC with no respiratory variation, and a variation of 25%/40% across the mitral/tricuspid valve inflow signals, respectively, during respiration [104]. Finally, echocardiography is used in the guidance of pericardiocentesis, for insertion of the drain and guidance of removal timing.

This is of interest as the cancer population is at an increased risk of a malignant pericardial effusion, particularly in advancing disease. As previously discussed in the anti-cancer treatment subsection on radiotherapy, there is an established relationship between radiotherapy and various pericardial diseases (e.g., calcific pericardial constriction). Myocardial involvement (e.g., for those on immune checkpoint inhibitor therapy) is suggested by echocardiography by regional wall motion abnormalities, global myocardial dysfunction, and/or pericardial effusion.

### 6.6. Cardiac Source of Embolism

As discussed in previous Sections, a major overarching side effect of anti-cancer treatment is ventricular systolic failure. Significant ventricular failure can lead to ventricular thrombi, which, when embolising, can cause organ ischaemia. It is well-established that heart failure and atrial fibrillation are closely associated [105], and patients in atrial fibrillation are known to develop thrombi in the left atrial appendage, again an ischaemic embolic risk. Primary atrial fibrillation (i.e., unrelated to ventricular dysfunction) can be associated with cyclophosphamide therapy. The cancer population is well recognised to carry a higher risk of venous thromboembolism.

A contrast-enhanced examination can establish left ventricular thrombi, leading to treatment by oral anticoagulation [106]. As previously discussed in the standard echocardiographic examination subsection, transthoracic echocardiography can assess for atrial septal defects, which are relevant in the treatment of embolic phenomena [107], for instance, in the establishment of intra-cardiac defects in a case of systemic embolism. The final subsection of this chapter will discuss the role of transoesophageal echocardiography, which includes an interrogation of the left atrial appendage for thrombotic phenomena.

### 6.7. Cardiac Masses

Echocardiography often discovers incidental masses that require further assessment. Embolic phenomena have been discussed in the previous subsection, and the following Section on cardiac CT and MRI delves into these phenomena in greater detail.

### 6.8. Transoesophageal Echocardiography

Transoesophageal echocardiography is an adjunct to transthoracic imaging and carries with it specific indications and standardised views [108]. It is completed ideally across three windows: transoesophageal, transgastric, and aortic. The specific disease processes for which it carries clinical use, that are particularly relevant to the population undergoing anti-cancer treatment for breast cancer, are listed in the table below (Table 8).

There are further indications to perform transoesophageal echocardiography; however, patients on anti-cancer treatment can be immunocompromised (with a higher risk of endocarditis), have a higher tendency to venous thromboembolism (relevant to cardiovascular source of embolism), and, finally, the anti-cancer treatments described in this review tend to associate with LV dysfunction, from which can occur secondary mitral regurgitation. The use of transoesophageal echocardiography can not only confer a diagnosis for these conditions, it can also guide treatment (e.g., size of vegetation requiring surgery, anatomy of intra-atrial defect, and suitability of mitral valve for edge-to-edge repair).

## 7. Cardiac CT

Cardiac CT has many indications that exceed this review. The following are specific topics within the cardio-oncology breast cancer population that will be of particular interest to the reader.

### 7.1. Coronary Artery Disease

Cardiac CT is a well-described indication for the screening for, and diagnostic of, coronary artery disease in several large multicentre prospective trials [109,110]. Beyond angiography of coronary vessels, cardiac CT can also accurately describe coronary artery calcium scoring [111]. A calcium score is a quantitative assessment of coronary calcium, where a unit of calcium within the coronary arteries is described as ≥1 mm^2^ in an area with a density of ≥130 Hounsfield units. A weighted sum of each calcification is taken, with the total figure labelled as the Agatston score. Beyond the description of coronary vasculature, the major benefit of coronary calcium scoring is in risk prediction of asymptomatic individuals [112].

In recent times, clinical concern has arisen regarding the sensitivity of treadmill exercise stress testing [113], giving rise to comparative studies between this and cardiac CT for the assessment of stable chest pain. Indeed, a focus arose to move away from a “rule-in” of coronary disease, to a “rule-out” focus by non-invasive testing. Cardiac CT evaluates all coronary segments > 1.5 mm in diameter for stenosis, including coronary artery bypass grafts. These stenoses (if present) are categorised under the CAD-RADS system in order of severity [114]. Cardiac CT further can describe high-risk plaque features, which are a clinically relevant finding in practice as they are associated with a higher prevalence of major adverse cardiac events [115]. These high-risk features include low-attenuation of plaque (as described in Hounsfield Units) and evidence of vessel remodelling.

Thanks to these developments, the cardiology community can now feel confident in CT as a more accurate method of assessment when compared to treadmill exercise stress testing. Specifically, cardiac CT reduces the rate of myocardial infarction, increasing the performance of invasive coronary angiography and revascularisation [116]. Two landmark prospective studies supportive of the use of cardiac CT for prognostic improvement of patients with chest pain are the PROMISE trial [117] and the SCOT-HEART trial [118]. These trials have been reflected through the European and American guidelines with cardiac CT a first-line test in evaluation of stable chest pain. Although not specific to the cardio-oncology patient population, one cannot document an overview of cardiac CT in stable chest pain without referring to these trials. In essence, the cardio-oncology population should be approached in a similar fashion to the general population, in the context of the anti-cancer treatment that they are receiving.

### 7.2. Cardiac Masses

Cardiac masses can be thought of as three distinct categories: benign tumours, neoplastic tumours, and non-neoplastic masses. Cardiac CT can provide additional information regarding intra/extra-cardiac masses. Most cardiac tumours (c 75%) [119] are benign, with myxoma, lipoma, and fibroelastoma making up half of all cardiac tumours. Malignant tumours are 20–40% more likely to be metastatic disease (commonly lung, breast, and melanoma primaries) than a primary cardiac tumour, which, if present, are most commonly angiosarcoma, rhabdosarcoma, and mesotheliomas. Only 2% of primary cardiac tumours are diagnostic of lymphoma. Examples of non-malignant masses are thrombi, calcification, vegetations (further described in the Section regarding infective endocarditis), pericardial cysts, hypertrophy, lipomatous changes, or artefact/normal variants. The location of mass is important also, as demonstrated in the following table (Table 9).

Cardiac CT has several facets to improve diagnostic accuracy at acquisition, increasing field of view, triphasic contrast protocols for right-sided masses, and the use of non-contrast CT acquisition to assess calcification, and differentiate fat and thrombus [120]. Benign tumours tend to be smooth, fat-rich, and usually lack contrast enhancement. This is in comparison to malignant masses, which have irregular borders, are calcific, and demonstrate arterial phase enhancement suggestive of vasculature. It is evident that it has a large scope of practice within the cardio-oncology population, and could help define cardiac metastases from a breast primary tumour.

### 7.3. Pericardial Disease

The pericardium is an avascular structure that normally contains 15–50 mL of clear fluid around the heart. Cardiac CT can assess for pericardial effusion and pericarditis [120]. Pericardial effusions appear as a low-attenuation, non-enhancing fluid accumulation. CT can describe its extent, presence of loculation, and suggest evidence of haemodynamic compromise (collapse of cardiac champers, flattening of intraventricular septum). Pericarditis is demonstrated as a smooth thickening with enhancement post-contrast and a variable amount of pericardial effusion. Chronic pericarditis is suggested by calcific changes, with constrictive pericarditis predicted by a thickness of >4 mm, enlarged atria, dilated inferior/superior vena cava and hepatic veins, and ascites/pleural effusion. Often, in clinical practice, if a significant effusion is identified at CT acquisition, urgent correlation via echocardiography (see subsection above) is indicated to further assess for haemodynamic compromise. Pericardial calcific disease requires correlation with the patient’s clinical status, and further testing if considering pericardiectomy (e.g., simultaneous left and right heart catheterisation).

### 7.4. Valvular Heart Disease

In the realm of cardio-oncology, cardiac CT has particular relevance in the work-up of severe aortic and mitral valve disease, especially when the severity of uncorrected valvular heart disease precludes systemic anti-cancer treatment. Additionally, as described in the final Section on extra-cardiac findings, as transcatheter fixation of these valves becomes increasingly prevalent, pre-procedural CT scanning can identify an incidental cancer process. Cardiac CT can describe bicuspid aortic valves, quantify stenosis via the Agatston score, and assess an associated aortopathy [121]. It is a key player in procedural planning for transcatheter aortic valve implantation, establishing aortic and peripheral anatomy, and clarifying the implantation projection angle [122].

In significant mitral valve disease, cardiac CT can reliably assess the mitral apparatus and adjacent structures prior to a transcatheter replacement. It is vital for mitral annular assessment and ‘neo’ left ventricular outflow tract measurement prior to transcatheter procedures [123]. At acquisition, cardiac CT can describe the papillary muscles, chordae tendinea, and the posterior/anterior mitral valve leaflets. The leaflets can be reformatted to create the ‘en face’ view, identify each of its scallops, and measure the mitral annular circumference [124]. Cardiac CT can interrogate for predictors of left ventricular outflow obstruction prior to transcatheter replacement, and also plan the co-axial view for deployment of a transcatheter replacement device. This is significant given the cancer patient population’s increased risk of endocarditis when immunocompromised, and also within the geriatric cancer population where dual processes are more common.

### 7.5. Left Atrium and Pulmonary Veins

Cardiac CT can describe the left atrium, pulmonary veins, and left atrial appendage. This is of particular clinical relevance in patients undergoing ablation for atrial fibrillation and appendage occlusion for oral anticoagulation intolerance [125,126]. It can carefully assess the shape and dimension of the appendage (types being Windsock, Cactus, Cauliflower, and Chicken Wing), and using delayed-phase imaging it is a valuable alternative to transoesophageal echocardiography for thrombus in the appendage. Post-procedurally it is a highly sensitivity modality to assess for peri-device leak, and can assess for thromboses. An accurate description of the anatomy in the atrium and pulmonary veins can allow for safe and effective catheter manipulations, and cardiac CT can detect complications after ablation for atrial fibrillation (e.g., atrio-oesophageal fistula and pulmonary vein stenosis). The interface of atrial fibrillation and heart failure was touched upon previously in this review, and the breast cancer patients in this situation may be receiving cyclophosphamide or other agents known to depress the ejection fraction, or be of an advanced age.

### 7.6. Infective Endocarditis

Cardio-oncology patients may often be immunocompromised by systemic anti-cancer treatment. Therefore, they are at a higher risk of native and prosthetic endocarditis, which includes cardiac device-related infective endocarditis. Cardiac CT has particular use where transoesophageal echocardiography is inconclusive [127], and can be combined at imaging with nuclear imaging (see Section 9.3). Cardiac CT is superior to TOE for the assessment of complications from endocarditis (e.g., abscess, fistula, and pseudoaneurysm), and non-cardiac-dedicated whole-body CT can assess for an embolic source and include angiography for mycotic aneurysms [128]. Vegetations themselves appear as mobile hypodense masses on cardiac CT, usually attached to a valve, device lead, or mural endocardium [129], are associated with regurgitant jets, and have no alternative anatomical explanation.

### 7.7. Extra-Cardiac Findings

A cardiac disease process need not always follow an oncological diagnosis in cardio-oncology. Indeed, it may be the inverse, where an individual undergoing evaluation/treatment for cardiac disease is found to have an oncological process. The use of Cardiac CT is becoming increasingly prevalent, so the topic of extra-cardiac findings is one of great relevance. The overall prevalence is c. 40% of extra-cardiac findings at CT, with 15% clinically significant [130]. These figures may be even higher in the senile aortic stenosis population undergoing CT angiography prior to transcatheter aortic valve implantation, where the field of view is wider and the average patient age is older (>70 to 75 years) than those under CT angiography for chest pain evaluation (typically <65 years old).

The most common area of extra-cardiac findings is the lung, followed by the abdomen, vessels, and mediastinum [129]. The following adapted table (Table 10) describes the prevalence of significant extra-cardiac findings of relevance to the cardio-oncology population [131]—the wide prevalence is in some instances indicative of the need for long-term data on this topic:

Pulmonary nodules are characterised by the Fleischner Societal guidelines—they are characterised by a solid/non-solid state of the nodule, and are felt to be at higher risk if there is a history of tobacco smoking, spiculation/emphysema/fibrosis also on CT, or family history of lung cancer. In mediastinal lymphadenopathy, a concomitant pneumonia suggests a benign origin, and alternative systems in this area include thyroid/thymic disorders. Hepatic nodules are typically benign < 10 mm, and most are cysts. However, metastases, when present, appear as solid, low-attenuation irregular nodules. Breast nodules, when present, can be malignant in c. 50%.

## 8. Cardiac MRI

Cardiac MR in this population of patients has particular use in pre-treatment risk stratification, early detection, and monitoring of cardiotoxicity, and the management of late effects. As such, the topics within this Section keep in mind the breast cancer population and how anti-cancer treatments may lead to cardiotoxic presentations. This is by no means an all-encompassing overview of cardiac MRI, but it serves to give a sense of the role it can play in this cohort.

### 8.1. Chronic Coronary Disease

Cardiac MRI can establish ischaemia and viability in the myocardium, and thus guide revascularisation strategy. In this patient population, it has particular use in guiding percutaneous vs. surgical revascularisation for multivessel disease, for medical vs. percutaneous therapy for single vessel disease, and in the selection of chronic total occlusion revascularisation candidates.

A standard protocol would include cine acquisition in the long and short axis, followed by T1 mapping for tissue characteristics and gadolinium administration for abnormal myocardial uptake, followed by stress cine acquisition [132]. Stress imaging can be performed either via a pressor agent (e.g., dobutamine) or by a vasodilator (commonly adenosine, dipyridamole, or regadenosone). In dobutamine stress imaging, the target heart rate is (220—age (0.85)). Vasodilator stressors are weight-based infusions that range from 0.5 to 6 min, with a target of haemodynamic response (tachycardia, hypotension).

General principles of viability are related to myocardial function and thickness, detection of scar by T1 mapping and gadolinium, and contractile response to inotrope. The chance of myocardial recovery is proportional to the burden of scar [133], with an established definition of viability that is <25% transmurality of scar in the myocardium, and non-viability when this scar exceeds >50%, with 25–50% considered indeterminate. Given that T1 mapping has high specificity, but modest sensitivity [134], gadolinium scar assessment remains the key factor in this area.

### 8.2. Acute Coronary Disease

Cardiac MRI has a role in acute coronary syndrome similar to that in chronic coronary syndrome where indecision arises regarding the appropriateness of revascularisation. Beyond this, it serves a prognostic purpose, where it can assess for microvascular obstruction and intramural haematoma [135]. These prognostic features are assessed for via a mixture of gadolinium enhancement, and tissue characterisation via T1, T2, and T2 star imaging.

It has a role in the assessment of non-thrombotic acute coronary syndrome presentations—myocarditis (see further discussion below), embolic myocardial infarction, as well as myocardial infarction with non-obstructive coronary arteries (MINOCA) and stress-mediated cardiomyopathy [136]. Stress-mediated cardiomyopathy is suggested by pronounced oedema without a scar, whereas an embolic infarction is suggested by oedema and a subendocardial scar. Lastly, oedema and the myocardial salvage index are further validated parameters of prognosis after acute myocardial infarction at T1 and T2 mapping [137]. This is interpreted by the extent of oedema corresponding to the severity of injury at an average of 5–7 days after the infarction. Further, the assessment of oedema leads to increased diagnostic accuracy in the diagnosis of myocardial infarction.

### 8.3. Cardiomyopathies

Cardiac MRI has a unique ability in non-ischaemic cardiomyopathy to characterise tissue. It can distinguish myocarditis, amyloidosis, Chagas disease, sarcoidosis, Fabry disease, and hemochromatosis [138]. Within its protocol, it can include a stress assessment (see chronic coronary disease, Section 8.1) to rule out an ischaemic aetiology. Beyond diagnostic information, it can infer prognostic information. Late gadolinium enhancement [139] can guide diagnostic and prognostic assessment, where in MRI it can assess oedema (see Section 8.2, on acute coronary disease), and establish scar pattern [140]. Typically, non-ischaemic scar/fibrosis is mid-wall in location, whereas an ischaemic origin is subendocardial. It has many other areas of utility: hypertrophic myocardial disease, infiltrative, arrhythmic, and storage diseases, and in systemic inflammatory processes.

### 8.4. Myocarditis

Related to immune checkpoint inhibitors, myocarditis is diagnosed by either clinical or pathohistological methods. Endomyocardial biopsy is not within procedural risks, and depending on the location of inflammation may be missed/non-reachable at biopsy. Clinical diagnosis via biomarkers and non-invasive imaging provides an alternative. A cardiac MR diagnostic for features of acute myocarditis is considered a major criterion in current European cardio-oncology guidelines [1] and is recommended as staged imaging during the recovery phase. The updated Lake Louise criteria [141] suggest the main criteria of myocardial oedema (on T2 mapping) and non-ischaemic myocardial injury (via abnormal T1 mapping, extracellular volume, or late gadolinium enhancement). Further, there are supportive criteria in the form of pericarditis (notable via effusion at cine acquisition, or abnormal late gadolinium enhancement or T1/T2 mapping) or systolic left ventricular dysfunction (by regional or global wall motion abnormalities).

### 8.5. Pericardial Disease

Typically, as previously described in other Sections, echocardiography is the most common initial test to diagnose pericardial disease, and CT imaging gives pertinent advice regarding calcification. Cardiac MR can not only diagnose an effusion at acquisition, but can be suggestive of acute pericarditis by the presence of inflammation as enhancement at imaging [142]. Due to its high image quality, cardiac MR can describe features such as loculation that may not always be evident from echocardiography imaging, or involvement of the myocardium. In recurrent pericarditis, cardiac MR is associated with a reduction in steroid use, pericardiocentesis, and overall recurrence [143]. Cardiac MR can describe typical findings in constrictive pericarditis (dilated atria, septal shift towards the left ventricle during inspiration) in a superior fashion to cardiac CT, as an adjunctive test to echocardiography, which remains the first line [144].

### 8.6. Valvular Heart Disease

A discussion was previously had in the subsection regarding cardiac CT and valvular heart disease regarding the interplay in the senile population of severe valvular pathology that may preclude systemic anti-cancer treatment, and the diagnosis of a malignancy via the non-invasive work-up of valvular heart disease progressing towards correction. As described in the subsection on radiotherapy, there is a relationship between radiotherapy and left-sided heart valve calcification. Cardiac MRI, whilst not a key component in this situation, can describe the myocardium, additional pathology, right-sided heart valves, and volumetric analysis of regurgitant jets. In cancer therapy-related cardiovascular toxicity, primary valvular conditions are uncommon, and predominantly are assessed by ultrasound and cardiac CT. As such, the discussion of cardiac MR will remain limited in this review.

### 8.7. Cardiac Masses

The prevalence of different cardiac masses, and the importance of location related to mass type, is discussed in the Section on cardiac masses within cardiac CT. However, cardiac MR has unique qualities in the assessment of tissue characteristics via T1 and T2 mapping, fat saturation sequences, determining intra- vs. extra-cardiac involvement, and patterns of gadolinium enhancement [145].

## 9. Nuclear Cardiology

Nuclear Imaging is undertaken when a radiopharmaceutical that accumulates in the heart is assessed for proportionality to myocardial flood flow, innervation, metabolism, and contractile function. Typical radiopharmaceuticals that are used are fluorodeoxygluclose (FDG), ammonia, rubidium chloride, sestamibi, tetrofosmin, and MIBG. These agents are associated with single-photon computed emission tomography (SPECT) and positron emission computed tomography (PET), and vary in radiation dose and the length of rest and stress protocol. Common clinical applications of SPECT are for ATTR-amyloidosis, evaluation of ischaemic heart disease, and ejection fraction (via multi-gated acquisition scanning, i.e., MUGA). Common clinical applications of PET are for viability, innervation, and amyloidosis. The following subheading reviews common indications of relevance to cardio-oncology.

### 9.1. Ischaemic Heart Disease

This evaluation is commonly undertaken by the use of vasodilators with a SPECT protocol—the vasodilators, namely, adenosine, dipyridamole, and regadenoson [146]. A protocol is undertaken at stress, and then at rest (after the vasodilator has worn off), to create a polar plot of myocardial perfusion images [147]. These are a series of images on the short and long axis. Artificial intelligence can calculate an ejection fraction and calcium score as required. An absence of ischaemic, a reversible or irreversible perfusion defect (i.e., established area of infarction), will be described. High-risk features include large perfusion defects (>15% of the myocardium), transient LV cavity dilatation with stress, drop in LVEF with stress, and an abnormal myocardial blood flow reserve. An established drawback is that of balanced ischaemia [148], often inferred by a high calcium score, but a negative perfusion study. PET is recognised to be superior to SPECT with better image quality and an ability to create myocardial flow reserves [147].

### 9.2. Non-Ischaemic Myocardial Disease

Nuclear imaging can describe non-ischaemic cardiomyopathy by the presence of normal myocardial perfusion imaging. The systolic and diastolic function can be established by any of MUGA scanning, SPECT, or PET. Phase analysis in SPECT can describe dyssynchrony, but a drawback of this form of imaging is an inaccurate assessment of RV function.

### 9.3. Inflammation and Infection Imaging

FDG-PET imaging has some indication in the multi-modal approach in this area [149]. This includes the assessment of a potentially infected prosthetic valve, mycotic aneurysms (save intra-cranial), presence of septic emboli, and the method of systemic entry (dental abscess, colon cancer, deep-seated infection). Mathieu et al. [150] described the FDG aspects in favour of active endocarditis at PET: high-intensity uptake, persistence of the signal on non-attenuated-corrected images, heterogeneity of signal, and FDG signal located to typical regions for infection processes or suspicious aspects of other imaging modalities. Regarding possible CIED infection, nuclear imaging plays a key role. It is not a simple matter for re-do procedures in patients with CIED—each procedure heightens the risk of peri-procedural complications. The use of nuclear imaging [151] was highly effective in the work-up of this patient population.

## 10. Risk Stratification

A foundation principle here regarding CTR-CVT is that absolute risk depends on a patient’s risk at baseline, and can alter over time. Further risk should be understood in two ways: how likely it is to happen, and how severe will it be if it occurs [1]. Beyond this, the definition of risk is impacted by the type of treatment, environment, and patient factors. A preliminary tool has been devised by the cardio-oncology study group of the Health Failure Association of the European Society of Cardiology, with external validation [152,153]. This tool can be used in conjunction with general population risk score calculators (e.g., the SCORE2 or SCORE2-OP) to give an individual/clinician a sense of the patient’s general cardiovascular risk, independent of their cancer treatment.

In a general sense, the approach to overall baseline risk stratification prior to any cancer therapy involves a focused history regarding cardiovascular and cancer status, cardiovascular risk factors, and prior cancer treatment(s). This information is then coupled with a physical examination (with a particular focus towards valvular heart disease, arrhythmia, signs of congestive cardiac failure, or pericardial disease) and an electrocardiogram. If the patient has pre-existing cardiovascular disease or is at risk of CTRCD, then they should undergo echocardiography (including global longitudinal strain imaging) and have their cardiac biomarkers obtained; as, to the best of the treating clinicians’ knowledge, these ‘secondary prevention’ patients are at a higher risk than ‘primary prevention’ patients. In those with a non-diagnostic echocardiogram, a cardiac MRI should be considered as an alternative.

The HFA-ICOS risk tool separates patients by the planned systemic anti-cancer treatment (anthracycline, HER2, VEGF inhibitors, RAF/MEK tyrosine kinase inhibitors, multiple-myeloma therapies, multi-target kinase inhibitors for CML). It does not extend to radiotherapy or endocrine therapy. Patients are further risk stratified by a previous history of cardiovascular disease (ischaemic heart disease, cardiomyopathy, and arrhythmia), elevated baseline cardiac biomarkers, age, cardiovascular risk factors (dyslipidaemia, diabetes mellitus, chronic kidney disease), previous cardio-toxic anti-cancer treatments, and lifestyle risk factors (smoking and obesity). The patient, at this stage, is designated a risk level of low, medium, high, or very high. Frequency of ECG, cardiac biomarkers, transthoracic echocardiography, or a referral for further guideline advice are also suggested.

Risk assessment can be performed by an appropriate clinician, e.g., oncologist, surgeon or cardiologist, and in those with a high/very high risk, a review by a cardiologist should be undertaken prior to anti-cancer treatment with a discussion including the patient regarding the risk/benefit balance of anti-cancer treatment. The following table (Table 11) summarises the approach to baseline risk stratification in breast cancer patients receiving systemic anti-cancer therapy, and is abridged from the recommendations of the European Society of Cardiology guidelines. It is based on the anti-cancer treatments that are listed in Section 4, and it should be noted that treatment-specific evidence-based advice is not available for all treatments based on current evidence.

Radiotherapy-related cardiovascular risk should be defined by mean heart dose [154], which is estimated using the number of fractions and dose to heart per fraction. However, it should be noted that this is not a perfect metric and, for example, may underestimate the risk of a small amount of the myocardium receiving a high level of irradiation, so the suggestion is that discretion by the cancer team is used regarding a patient’s risk of radiotherapy-induced cardiovascular toxicity. The following table (Table 12) suggests the various risk categories a patient undergoing radiotherapy may be included within, and it is adapted from current European guidelines.

For patients undergoing surgery for breast cancer, a cardiac assessment is suggested by societal guidelines when a patient has a history of significant cardiovascular disease, symptomatic cardiovascular disease, a history of neoadjuvant possibly cardiotoxic chemotherapy, or a high/very high CVD risk according to the HFA-ICOS score.

There are suggested monitoring protocols for patients undergoing the above treatments, summarised in the following tables (Table 13, Table 14 and Table 15). First are anthracyclines (adapted from the ESC guidelines inclusive only of class 1 indications).

As apparent from the above tables, there is a gap in evidence for certain chemotherapeutical agents—fluoropyrimidines, platinum agents, CKDi 4/6, nucleoside analogues, HER2-specific TKA, taxanes, and alkylating agents. Fluoropyrimidine cardiac-induced toxicity often occurs at a short time interval to administration (usually within days). Although still with limited understanding, most patients who experience this side effect do not have a history of coronary artery disease [155]. No specific surveillance protocol is currently recommended, but CT coronary angiography or invasive angiography could be considered in asymptomatic patients and those with angina, to mitigate risk prior to treatment. The same extends to cyclophosphamide, where no specific radiomic or biological surveillance is advised, but an awareness of the association between it and atrial fibrillation and myocardial dysfunction. Platinum-based therapies do not have current guidance on the estimation of a patient’s baseline risk, and regarding monitoring, it is advised that a judicious eye be vigilant regarding chest pain events. Patients receiving Cyclin-Dependent Kinase 4/6 inhibitors should have an ECG at baseline, at day 14 of the first cycle, before the second cycle, and thereafter at dose increase or, if separate, at clinical indication. Endocrine therapy patients should be assessed similarly to the general population for cardiovascular risk at baseline (i.e., with SCORE2/SCORE2-OP) and at annual intervals. Patients receiving radiotherapy are recommended to undergo SCORE2/SCORE2-OP at baseline.

## 11. Prevention and Treatment of Cancer Therapy-Related Cardiovascular Toxicity

In general, regarding primary prevention, non-modifiable and modifiable risk factors should be approached in the same way as the general population, for example, smoking cessation, maintaining physical activity, and refraining from excess alcohol intake, anti-hypertensive, and anti-hyperglycaemic agents as appropriate. Drug–drug interactions for those on anti-cancer therapy agents are extensive and available in detail in the 2022 cardio-oncology European Society of Cardiology supplementary tables [1], for example, avoidance of concomitant QT-prolonging medications on those receiving Cyclin-Dependent Kinase 4/6 inhibitors. Discussed in the previous subsection was the role that monitoring plays in certain agents, and this is synergistic with the prevention and treatment approaches.

General advice extends to endocrine therapy, where blood pressure and lipid profile should be periodically measured and treated to target, as per societal guidelines in cardiovascular disease prevention. Patients on aromatase inhibitor therapy should be encouraged regarding physical activity and a healthy diet to mitigate their risk of coronary artery disease. Patients receiving tamoxifen therapy should be discouraged from smoking to reduce the risk of venous thromboembolism.

Prevention of radiotherapy-induced cardiovascular toxicity primarily focuses on reducing exposure of the heart to radiotherapy. As in general radiology, involving exposure to ionising radiation, the principle of ‘ALARA’ (as low as reasonably achievable) should be used—specifically analysing the requirement of radiotherapy in the first instance, when indicated using the lowest volume and dose of radiotherapy, and the utilisation of modern techniques to attenuate exposure of the heart. These include respiratory-gated techniques, and image-guidance and intensity-modulated photon technologies [156].

Alkylating agents should be recognised for their increased risk of long-term cardiovascular disease, taxanes for the risk of heart failure, and platinum therapies for vascular complications. The physician should tailor their approach to the patient taking these factors into account.

### Treatment

The following table (Table 16) demonstrates high-level evidence currently available in the cardiac pharmacotherapy treatment of patients receiving anti-cancer treatment [157,158,159,160,161,162,163,164,165,166,167].

Armed with the knowledge above, and coupled with lower-level evidence (observational studies, consensus documents, etc.), some management plans for treatment-related cardiotoxic events have been suggested (Table 17 and Table 18). It should be noted that no randomised prospective evidence regarding the role of SGLT2 inhibitors is currently available.

The anthracycline-related CTR-CVT approach is outlined in the following table, and is abridged from current guideline recommendations. Heart Failure therapy is a generic term for pharmacological (ACE inhibitor, beta-blocker, mineralocorticoid antagonist, SGLT2 inhibitor, and neprilysin inhibitor class agents) and non-pharmacological interventions (cardiac rehab, PCI, device therapy, arrhythmia ablation, etc.). Please note the difference in recommendation levels in mild asymptomatic CTRCD. Beyond these recommendations, if the anthracycline therapy net benefit is still felt appropriate by the MDT, the use of a reduced anthracycline dose, use of liposomal anthracycline, or pre-treatment with dexrazoxane all remain options. In these patients, closer clinical monitoring of every 1–2 cycles is recommended.

HER2-related CTR-CVT approach is again listed in the following table. Please note the lower level of recommendation in mild and moderate asymptomatic CTRCD.

ICI Myocarditis should be managed as an inpatient in a hospital setting with ECG monitoring, with cessation of the ICI. It should be separated as fulminant or not (haemodynamic instability, need for non-invasive/invasive ventilation, advanced heart block, or ventricular arrhythmia). Methylprednisolone once daily intravenously should be administered over three days. If then recovered, the patient should be de-escalated to oral prednisolone 1 mg/kg/day and weaned by 10 mg/week until recovery. If steroid refractory, the patients should be commenced on second-line immunosuppression (e.g., mycophenolate, tocilizumab, IVIG, or plasma exchange). Patients with fulminant myocarditis should be managed in the intensive care unit with consideration given to mechanical circulatory support. In non-myocarditis toxic syndromes (e.g., atrial fibrillation, cardiac failure), the patient can be managed as per the general population, and the ICI can be continued.

Currently, we do not have high-level evidence to support an algorithmic approach to the other agents discussed in this introduction (radiotherapy, endocrine therapies, taxanes, fluoropyrimidines, platinum, alkylating, HER2 tyrosine kinase agents, and cyclin-dependent kinase 4/6 inhibitors). The management of CTR-CVT from these agents is often complicated by clinical confounders: thrombocytopenia, frailty, predisposition to thrombosis or bleeding, and need for staged surgery/procedures. A multidisciplinary discussion attempts to factor in these elements. For myocardial infarction (associated with carboplatin, nucleoside analogues, and fluoropyrimidines), the consensus is that when life expectancy exceeds six months, percutaneous coronary intervention is appropriate where it would palliate a patient’s symptoms [168]. Radial access, judicious use of heparin, and a baseline platelet count of over 30 are important factors. If strong suspicions of a correlation between anti-cancer agents and infarction are held, then alternative agents should be explored. For a stable patient with chronic coronary syndrome (radiotherapy-related), the work-up remains similar to the general population, with a discussion between senior physicians regarding the appropriateness of percutaneous coronary intervention. The same philosophy should apply to patients with heart failure syndrome secondary to the other anti-cancer treatments discussed in this review (cyclophosphamide, HER2 tyrosine kinase inhibitors, nucleoside analogues, and taxanes), in which the introduction of heart failure therapies and consideration of anti-cancer treatment cessation should be discussed.

Valvular heart disease (in this review related to previous radiotherapy treatment) should be treated in the same fashion as the general population. For those with infectious endocarditis secondary to immunocompromise, the European Society of Cardiology has thorough advice in this regard in their Infective Endocarditis Guidelines. Atrial fibrillation (related to cyclophosphamide or nucleoside analogues) should be managed in a rate vs. rhythm control approach, with the judicious interrogation of drug–drug interactions and a bleeding/thrombosis risk with regards to oral anticoagulation. In those unable to tolerate oral anticoagulation, left atrial appendage closure could be considered after a multidisciplinary discussion. Conduction disease in the context of previous radiotherapy treatment should be managed as per the general population. Venous thrombosis (related to endocrine therapies and carboplatin) should have a bleeding/thrombosis assessment made, and this should be discussed with the patient. With an indwelling venous catheter, 3 months is sufficient if the catheter is removed. NOACs are appropriate first-line agents, with low molecular weight heparin as an alternative. If the breast malignancy remains active (e.g., with metastatic disease), then continuation of anticoagulation beyond 6 months is recommended (if no venous catheter in situ). Pericardial disease related to gemcitabine is caused by underlying capillary leak syndrome and is responsive to glucocorticoid therapy. Lastly, in stroke syndromes (e.g., in patients on endocrine therapy or with previous neck radiotherapy leading to carotid stenosis), a case-by-case approach should be formed, with a multidisciplinary discussion as appropriate.

## 12. Long-Term Follow-Up

The patients who would benefit from a long-term follow-up include those with a high risk of cardiotoxicity, either by HFA-ICOS risk score [152] or who received agents known to have long-term cardiovascular toxic effects [169], e.g., doxorubicin dose ≥ 250 mg/m^2^, radiotherapy > 15 Gy mean heart dose, or doxorubicin ≥ 100 mg/m^2^ with radiotherapy of 5–15 Gy mean heart dose. Additionally, those who suffered either moderate or severe CTR-CVT [170] would benefit from a long-term follow-up—e.g., CTRCD as described in previous Sections, ICI-related myocarditis, and severe vascular pathologies (acute coronary syndromes).

For breast cancer patients treated with doxorubicin, there is a linear relationship with cumulative dose, with a cohort up to 12 years post-treatment more likely to have impaired left ventricular ejection fraction, global longitudinal strain, and natriuretic peptides [171]. Radiotherapy effects tend to be seen 5–10 years after treatment for breast cancer [172], and its clinical effects have been described in the preceding subsections. The following table (Table 19) describes how this follow-up may be undertaken, and it is adapted from current European guidelines. High/very high has been described in the previous paragraph, and here moderate includes moderate baseline risk, doxorubicin dose 100–249 mg/m^2^, radiotherapy mean heart dose of 5–15 Gy, or doxorubicin ≥ 100 mg/m^2^ with radiotherapy of <5 Gy mean heart dose. Low risk includes low baseline risk, mild CTRCD during treatment with resolution before completion, doxorubicin dose < 100 mg/m^2^, and radiotherapy < 5 Gy mean heart dose.

This approach should be coupled with patient education and cardiovascular risk factor management, which can be performed conjointly with primary care. Screening for coronary artery disease should be considered at 5 years after treatment in a high-risk patient. If new symptoms develop, then these should be addressed at the discretion of the treating physician. At present, outside radiotherapy and anthracyclines, there is no evidence regarding CTR-CVT and the other anti-cancer agents discussed in this review. The current consensus from the European Society of Cardiology is that asymptomatic patients who received potentially cardiotoxic therapy should have an ECG, annual review, and natriuretic peptides annually for 5 years, with a risk re-stratification after this to assess the need for a long-term follow-up.

## 13. Conclusions

This review aims to provide an in-depth assessment regarding the interface between breast cancer and treatment-induced cardiovascular toxicity. Cardiac biomarkers, echocardiography, cardiac CT, cardiac MRI, and nuclear medicine all have a role to play as diagnostics dependent on the clinical situation. Table 20 references the initial table in this review describing various toxicity definitions, and is at this point adapted to demonstrate how the different modalities can be utilised. Multiple gaps in the evidence remain regarding certain anti-cancer agents in terms of a patient’s cardiovascular risk prediction and appropriate monitoring during treatment. However, in those with a higher level of evidence, strategies remain for diagnosis and management of cardiovascular toxicity. Further studies regarding these anti-cancer agents and cardiovascular toxicity are warranted, as are randomised studies involving SGLT2 inhibitors.

## Figures and Tables

**Table 1:** Overview of Cancer therapy-related cardiovascular toxicity and cardiac dysfunction.

CTR-CVT	CTRCD
Heart Failure - See CTRCD opposite. - Most commonly encountered with HER2 agents (Trastuzumab), Anthracyclines, VEGF treatment, and MEK inhibitor Cobimetinib.	Symptomatic CTRCD - Mild (mild heart failure symptoms). - Moderate (need for outpatient intensification of heart failure therapy). - Severe (hospitalisation with heart failure). - Very Severe (need of mechanical or inotropic support, or heart transplant).
Myocarditis - By clinical or pathohistological diagnosis. Mostly associated with Immune Checkpoint Inhibitors. Vascular Toxicity - Symptomatic (stroke, myocardial infarction, chronic coronary syndrome, claudication from peripheral arterial disease). - Asymptomatic (coronary/carotid/peripheral artery disease). - Associated with platinum agents. - Venous thromboembolic disease. Most commonly associated with immunomodulatory drugs (e.g., Lenolidomide). Arterial Hypertension - Cancer therapy holding threshold is ≥180/110 mmHg. - Hypertensive emergency described as in general population. - Most commonly encountered with Vascular Endothelial Growth Factor Tyrosine Kinase Inhibitors, 3rd generation BCR-ABL TKI (e.g., Ponatinib), proteosome inhibitors (e.g., Carfilzomib), monoclonal antibodies (e.g., Daratumumab), RAF inhibitors (e.g., Dabrafenib), MEK inhibitors (e.g., Binimetinib), Androgen metabolism inhibitors (e.g., Abiraterone) and ALK inhibitors (e.g., Lorlatinib). Cardiac Arrhythmia - Supraventricular. - Ventricular. - Atrial Fibrillation most commonly associated with alkylating agents (e.g., cyclophosphamide and melphalan). Prolongation of corrected QT interval - >500 ms by Fredericia criteria. - Most commonly seen with the 2nd generation BCR-ABL TKI nilotinib. Pericardial Disease - Same definition as general cardiology population. Valvular Heart Disease - Same definition as general cardiology population.	Asymptomatic CTRCD - Mild (LVEF ≥ 50% and new decrease in global longitudinal strain >15% and/or new rise in cardiac biomarkers). - Moderate (LVEF reduction ≥ 10% to 40–49%, or LVEF reduction < 10% to 40–49% with new reduction in global longitudinal strain >15% or new rise in cardiac biomarkers). - Severe (LVEF reduction to <40%).

**Table 2:** Typical Anti-Cancer Agents for Breast Cancer.

Chemotherapy Agent	Mechanism of Action
Carboplatin	Forms intracellular platinum complexes inhibiting DNA synthesis
Cyclophosphamide	Alkylating agent: leads to cross-linking of DNA inhibiting protein synthesis
Fluoropyrimidines (5-Fluorouracil/Capecitabine)	Inhibits processing, maturation, and modification of RNA
Taxanes (Paclitaxel, Docetaxel)	Inhibits mitosis and induces apoptosis
Anthracyclines (Doxorubicin, Epirubicin)	Poison topoisomerase causing programmed cell death
Immune Checkpoint Inhibitors (Pembrolizumab, Atezolizumab)	Blocks checkpoint proteins from binding with partner proteins, thus allowing T cells to kill cancer cells
Cyclin-dependent Kinase 4/6 inhibitors (Ribociclib)	Through inhibition of this pathway, inhibiting tumour cell proliferation
HER2-specific Tyrosine Kinase Inhibitor (Tucatinib, Lapatinib)	Inhibits phosphorylation of HER2 and HER3, affecting downstream MAPK, AKT and cell proliferation
Nucleoside Analogues (Gemcitabine)	Incorporation of dFdCTP into DNA leading to cell death

**Table 3:** Early Receptor-Positive Breast Cancer.

Receptor-Positive	HER2	Triple-Negative
Premenopausal: Luminal A/B (stage 1–3) = Tamoxifen Postmenopausal = Tamoxifen followed by AI High-risk Luminal A/all luminal B—neoadjuvant CT. If stage 3 or high-risk stage 2, then include gBRCA1/2 testing (include CDK4/6i treatment if positive)	Determined by >T2 or node-positive disease Involves surgery, radiotherapy (if indicated), and mixture of trastuzumab, chemotherapy (e.g., paclitaxel) and endocrine therapy (if receptor-positive)	Determined by tumour and nodal status. If >T1c stage, then for neoadjuvant taxane + platinum therapy (consider pembrolizumab). Then, surgery/radiotherapy (performed first is stage < T1c). Clarify gBRCA1/2 (including wild-type). Further treatments include CDK4/6i, ICI and capecitabine.

AI = aromatase inhibitor, CT = chemotherapy.

**Table 4:** Metastatic HER2-Positive Breast Cancer.

1st Line	2nd Line	3rd Line
(1) HR-positive Trastuzumab (if chemotherapy CI) and ET Or Trastuzumab + Docetaxel for over 6 cycles (if no CI to chemotherapy), followed by trastuzumab + ET until progression (2) HR-negative Trastuzumab until progression (if chemotherapy CI) Or Trastuzumab + Docetaxel for over 6 cycles (if no CI to chemotherapy), followed by trastuzumab + ET until progression	(1) If no/stable BM Trastuzumab deruxtecan (2) If active BM Local intervention not required: Tucatinib + Capecitabine + Trastuzumab Or Trastuzumab deruxtecan If local intervention required: 1–10 BM + favourable features then consider SRT ± resection; >10 then consider whole brain radiotherapy	(1) If no/stable BM Any of: Lapatinib + Capecitabine, Lapatinib + Trastuzumab, or chemotherapy + Trastuzumab (2) If active BM Local intervention not required: Any of: Lapatinib + Capecitabine, Lapatinib + Trastuzumab, or chemotherapy + Trastuzumab If local intervention required: 1–10 BM + favourable features then consider SRT ± resection; >10 then consider whole brain radiotherapy

HR = hormone receptor, CI = contraindicated, ET = endocrine therapy, BM = brain metastases, SRT = stereotactic radiotherapy.

**Table 5:** Metastatic Oestrogen-Positive.

1	2	3	4	5	6
Endocrine therapy + CDK 4/6i Or Chemotherapy (if imminent organ failure) with trastuzumab deruxtecan after 1st line (if HER2 low)	-> (progressive disease)	Decide progression-free survival and if imminent organ failure	If no organ failure, then defer to genetics (PIKC3Am, ESR1m, BRCA/PALB2m) to guide endocrine therapy. If organ failure or short survival proceed to column on far right.	-> (progressive disease	Trastuzumab deruxtecan + Chemotherapy (in low HER2) Or Sacituzumab + Chemotherapy (if no HER2)

Chemotherapy = any of anthracyclines, taxanes, capecitabine, platinum agents.

**Table 6:** Metastatic Triple-Negative Breast Cancer.

PD-L1-Positive	gBRCAm-Positive	gBRCAm Wild-Type-Positive
Atezolizumab + Paclitaxel Or Pembrolizumab + chemotherapy Then if progression Sacituzumab or chemotherapy Then if progression Trastuzumab deruxtecan (if HER2 low) or capecitabine	Platinum chemotherapy (taxane alternative but less desirable option) Then if progression Sacituzumab or chemotherapy Then if progression Trastuzumab deruxtecan (if HER2 low) or capecitabine	If no IOF Taxane or anthracycline monotherapy If IOF Anthracyline + taxane Or Taxane + Bevacuzimab Or Capecitabine +Bevacuzimab Then if progression Sacituzumab or chemotherapy Then if progression Trastuzumab deruxtecan (if HER2 low) or capecitabine

Chemotherapy = paclitaxel or gemcitabine/carboplatin, IOF = imminent organ failure.

**Table 7:** The Standard Echocardiographic Examination.

Stage	Element	Features
1	2D Echocardiography and M mode	Two-dimensional acquisition of parasternal (long and short axis and right ventricular outflow view, including diameter of LVOT), apical (2, 3, 4 and 5 chamber), subcostal and suprasternal windows (consider right parasternal window). M mode: Intraventricular septal diameter, LV internal diameter (diastole), posterior wall thickness (parasternal long axis) Aortic root and left atrium (parasternal long axis). Through mitral valve (parasternal long axis). Through RV free wall for TAPSE (apical 4-chamber view). Through IVC through inspiration/expiration for respiratory variation.
2	Doppler Echocardiography	Aortic Valve: Continuous wave Doppler in multiple views (apical, suprasternal, right parasternal), measure peak gradient, trace velocity time integral for mean gradient, and trace LVOT VTI for valve area and cardiac output. If a regurgitant jet is present then assess pressure half-time in a 3- or 5-chamber view by tracing a continuous Doppler jet (important that Doppler is coaxial), and measure deceleration time. Mitral Valve: Either continuous or pulsed wave Doppler in apical view for E and A wave, trace velocity curve for VTI and mean gradient. If stenosis is present, measure pressure half-time for valve area. In regurgitation, assess envelope (complete envelope indicating severe). Tricuspid Valve: Either continuous or pulsed wave Doppler in apical view on ventricular side if concern for stenosis, and trace VTI if present. Place on atrial side and repeat for regurgitant jet. In regurgitation assess envelope (complete envelope indicating severe). Pulmonary Valve: Use left parasternal or subcostal view. Other: In suprasternal view, if aortic regurgitation is present, then assess for diastolic flow reversal, assess pulmonary venous flow in apical 4 chamber with pulsed wave Doppler.
3	Colour-Flow Doppler	Aortic: In stenosis flow, acceleration can help different locations of stenosis (valvular vs. sub/supravalvular). In regurgitation, describe timing (early diastole or holo-diastolic), jet width, measure vena contracta, and calculate EROA and regurgitant volume. Mitral: PISA method can calculate mitral valve area in stenosis. In regurgitation, comment on atrial area covered by jet, measure vena contracta and PISA. Tricuspid: PISA method can calculate mitral valve area in stenosis. In regurgitation, comment on atrial area covered by jet and vena contracta. Pulmonary: Appears as flow acceleration across the valve when visualised. Other: Across intra-atrial septum and intra-ventricular septum in apical view.
4	Tissue Doppler Imaging	As per manufacture settings in TDI mode, calculate Doppler over the septum and lateral wall for e’.
5	Flow-related calculations	Stroke Volume Cardiac Output Qp:Qs (if shunt present) Right atrial pressure Pulmonary Artery Systolic Pressure
6	Extra considerations	Atrial volume Strain Imaging Three-dimensional Echocardiography Contrast enhancement/Use of agitated medium

**Table 8:** Uses of Transoesophageal Echocardiography in the Breast Cancer Population.

Disease Process	Essential Imaging Features
Cardiovascular source of embolism	Imaging of the left atrial appendage inclusive of pulsed wave Doppler for inflow velocity, assessment of left atrium for spontaneous echo contrast, valvular assessment for vegetations and masses, ascending and descending aorta for mural thrombus, and intra-atrial septum for foramen ovale, septal defect/aneurysmal septum.
Infective Endocarditis (inclusive of prosthetic material)	Assessment of mitral valve in multiple cross sections, aortic valve in short/long axis, tricuspid in transgastric and oesophageal window inclusive of RVOT, and pulmonary valve in short axis. Important to include central venous catheter and intra-cardiac device leads as present.
Mitral Regurgitation	Description of mitral anatomy, mechanisms and origin of regurgitation, colour Doppler in left atrium to perform PISA, and left/right upper pulmonary vein venous flow.

**Table 9:** Cardiac Masses.

Location	Most Commonly Found Masses
Right Atrium	Thrombus, Eustachian Valve, Myxoma, Lipoma
Left Atrium	Myxoma, Lipomatous Hypertrophy, Thrombus, Coumadin Ridge
Right Ventricle	Thrombus, Angiosarcoma, Rhabdomyosarcoma, Metastasis
Left Ventricle	Thrombus, Metastasis, Fibroelastoma
Pericardium	Pericardial Cyst, Metastasis
Valves	Thrombus, Vegetation, Papillary Fibroelastoma, Calcification

**Table 10:** Extra-Cardiac Findings.

Extra-Cardiac Finding	Prevalence
Suspicious Pulmonary Nodule	0.4–16.5%
Pleural effusion	0.1–4%
Mediastinal Lymphadenopathy	0.1–2.3%
Indeterminate Hepatic Nodule	0–2.3%
Pulmonary Embolism	0–1.9%
Breast nodules	0–0.6%

**Table 11:** Baseline Risk Stratification by Systemic Anti-Cancer Agent.

Agent	Risk Level	TTE	NP	cTn
Anthracycline	High/Very High	Class 1	Class 1	Class 1
	Medium	Class 1	Class 2a	Class 2a
	Low	Class 1	Class 2a	Class 2a
HER2	High/Very High	Class 1	Class 1	Class 1
	Medium	Class 1	Class 2b	Class 2b
	Low	Class 1	Class 2b	Class 2b
Fluoropyrimidines	If previous CVD	Class 1	-	-
ICI	High/Very high	Class 1	Class 1	Class 1
	All others	Class 2b	Class 1	Class 1
Cyclophosphamide	-	-	-	-
Taxanes	-	-	-	-
Carboplatin	-	-	-	-
Radiotherapy (to a volume involving the heart)	If previous CVD	Class 2b		
CDKi 4/6	-	-	-	-
Nucleoside Analogue	-	-	-	-
HER2-specific TKI	-	-	-	-

NP = natriuretic peptides, TTE = transthoracic echocardiography, cTn = cardiac troponin, ICI = immune checkpoint inhibitors, CKDi = cyclin-dependent kinase inhibitors, TKI = tyrosine kinase inhibitor, CVD = cardiovascular disease.

**Table 12:** Risk by radiotherapy treatment.

Risk	Features
Low	<5 Gy mean heart dose
Moderate	5–15 Gy mean heart dose Or <5 Gy mean heart dose, and cumulative doxorubicin dose ≥100 mg/m^2^
High	>15–25 Gy mean heart dose Or 5–15 Gy mean heart dose, and cumulative doxorubicin dose ≥100 mg/m^2^
Very High	>25 Gy mean heart dose Or >15 Gy mean heart dose, and cumulative doxorubicin dose ≥100 mg/m^2^

Gy = Gray.

**Table 13:** Anthracycline Monitoring Protocol.

	Type	Baseline	Cycle 1	Cycle 2	Cycle 3	Cycle 4	Cycle 5	Cycle 6	3 m	12 m
Low	ECG	Class 1	-	-	-	-	-	-	-	-
	TTE	Class 1	-	-	-	-	-	-	-	Class 1
	NP/cTN	-	-	-	-	-	-	-	-	-
Moderate	ECG	Class 1	-	-	-	-	-	-	-	-
	TTE	Class 1	-	-	-	-	-	-	-	Class 1
	NP/cTN	-	-	-	-	-	-	-	-	-
High/Very high	ECG	Class 1	-	-	-	-	-	-	-	-
	TTE	Class 1	-	Class 1	-	Class 1	-	Class 1	Class 1	Class 1
	NP/cTN	Class 1	Class 1	Class 1	Class 1	Class 1	Class 1	Class 1	Class 1	Class 1

3 m = 3 months after treatment, 12 m = 12 months after treatment, TTE = transthoracic echocardiography, NP = natriuretic peptide, cTN = cardiac troponin, ECG = electrocardiogram.

**Table 14:** HER2 Agent Monitoring Protocol.

Risk	Type	Baseline	3 m	6 m	9 m	12 m	3 m Post	12 m Post
Low and Moderate	ECG	Class 1	-	-	-	-	-	-
	TTE	Class 1	Class 1	Class 1	Class 1	Class 1	-	Class 1
	NP/cTn	-	-	-	-	-	-	-
High and Very High	ECG	Class 1	-	-	-	-	-	-
	TTE	Class 1	Class 1	Class 1	Class 1	Class 1	Class 1	Class 1
	NP/cTN	Class 1	-	-	-	-	-	-

3 m = 3 months into treatment, 6 m = 6 months into treatment, 9 m = 9 months into treatment, 12 m = 12 months into treatment, 3 m post = 3 months after treatment completion, 12 m post = 12 months after treatment completion.

**Table 15:** Immune Checkpoint Inhibitor Monitoring Protocol.

Risk	Type	Baseline	Cycle 2	Cycle 3	Cycle 4	Every 3C	Every 6–12 m
Low	CV Ax	Class 1	-	-	-	Class 1	-
	ECG	Class 1	-	-	-	-	-
	TTE	-	-	-	-	-	-
	cTN	Class 1	-	-	-	-	-
	NP	Class 1	-	-	-	-	-
High	CV Ax	Class 1	-	-	-	Class 1	Class 1
	ECG	Class 1	-	-	-	-	Class 1
	TTE	Class 1	-	-	-	-	-
	cTN	Class 1	-	-	-	-	-
	NP	Class 1	-	-	-	-	Class 1

CV Ax = cardiovascular assessment.

**Table 16:** High-level evidence of anti-cancer-induced cardiotoxicity pharmacology.

PI	Type	Agent	Cancer	Outcome
Kalay	RCT (N = 50)	Carvedilol	Anthracycline	In Carvedilol vs. placebo, carvedilol arm mean LVEF remained unchanged (*p* = 0.3), placebo arm decreased from 68.9% to 52.3% at 6 months (*p* = 0.001)
Cardinale	RCT (N = 114)	Enalapril	High-dose chemotherapy	In enalapril vs. standard of care, no patients on enalapril had LVEF < 50%/decrease by 10%, whereas 43% of standard of care group had decrease in LVEF > 10%
Bosch	RCT (N = 90)	Enalapril and Carvedilol	Autologous HSCT	In medication vs. standard of care group, standard of care had a lower mean LVEF of 3.1% at 6 months (as measured by TTE and CMR)
Akpek	RCT (N = 83)	Spironolactone	AC for BC	Those on spironolactone had a more modest LVEF reduction (1.3%) than the placebo arm (14.4%)
Gulati	RCT (N = 130)	Candesartan or Metoprolol	AC for BC	Metoprolol arm had no effect on LVEF decrease; otherwise, decrease was 2.6% (placebo) and 0.8% (candesartan)
Pituskin	RCT (N = 94)	Perindopril or Bisoprolol	Tz for BC	No difference in primary endpoint (left ventricular end-diastolic volume)
Avila	RCT (N = 200)	Carvedilol	AC, Cycph, Paclitaxel	No difference in primary endpoint (>10% LVEF decline at 6 months)—placebo 13.%, carvedilol 14.5% (*p* = 1)
Cardinale	RCT (N = 273)	Enalapril	AC	Primary endpoint of elevated troponin was 23% in enalapril group vs. troponin-triggered cohort (26%)—*p* = 0.5
Guglin	RCT (N = 468)	Lisinopril + Carvedilol	Tz for HER2 BC	No difference in cardiotoxicity between study arms (drop in LVEF > 10%, or <5% to below 50%)
Shah	MA (9/771)	BB	Tz for HER2 BC	Post-chemotherapy LVEF was higher in BB group than placebo arm (3.84%, CI 95% 1.48–6.19)
Vaduganathan	MA (17/1984)	BB, MRA, ACEi/ARB	Ca treatment in those randomised to neurohormonal tx vs. placebo	NH treatment was associated with higher LVEF (1%, CI 95% 0.57–1.5)

RCT = randomised control trial, LVEF = left ventricular ejection fraction, HSCT = haematopoietic stem cell transplant, TTE = transthoracic echo, CMR = cardiac MRI, AC = anthracycline, BC = breast cancer, Tz = trastuzumab, Cycph = cyclophosphamide, MA = meta-analysis (stated here as number of studies/total amount of patients), CI = confidence interval, BB = beta blocker, MRA = mineralocorticoid receptor antagonist, ACEi = angiotensin-converting enzyme inhibitor, ARB = angiotensin receptor blocker, tx = treatment, NH = neurohormonal.

**Table 17:** Anthracycline-induced cardiac dysfunction management.

	1	2	4	5
Symptomatic CTRCD	Mild	MDT discussion	->	Heart Failure Therapy (1)
	Moderate	Interrupt	->	Heart Failure Therapy (1)
	Severe	Discontinue	->	Heart Failure Therapy (1)
Asymptomatic CTRCD	Mild	Continue + Monitor	NP Increase	ACEi/ARB + BB (2b)
			GLS decrease >15% or cTn increase	ACE/ARB + BB (2a)
	Moderate/Severe	Interrupt	->	Heart Failure Therapy (1)

CTRCD = cancer therapy-related cardiac dysfunction, MDT = multidisciplinary team, NP = natriuretic peptides, ACEi = angiotensin-converting enzyme inhibitor, ARB = angiotensin receptor blocker, BB = beta blocker, GLS = global longitudinal strain, cTn = cardiac troponin.

**Table 18:** HER2 therapy-induced cardiac dysfunction management.

	1	2	4	5
Symptomatic CTRCD	Mild	MDT discussion	->	Heart Failure Therapy (1)
	Moderate, severe or very severe	Interrupt + MDT	->	Heart Failure Therapy (1)
Asymptomatic CTRCD	Mild	Continue + Monitor	GLS decrease >15% or NP or cTn increase	ACEi/ARB + BB (2a)
	Moderate	Continue + Monitor (2a)	->	Heart Failure Therapy (1)
	Severe		->	Heart Failure Therapy (1)

**Table 19:** Method of Long-term Follow-up.

Risk	Annual Assessment	TTE
Low	Clinical exam, ECG, and natriuretic peptides each year	-
Moderate	Clinical exam, ECG, and natriuretic peptides each year	Transthoracic echocardiography every 5 years after treatment completion (2b)
High/Very High	Clinical exam, ECG, and natriuretic peptides each year	Transthoracic echocardiography 1, 3, and 5 years after treatment completion, and every 5 years after this (2a)

**Table 20:** Incorporating diagnostics into cancer therapy-related cardiovascular toxicity and cardiac dysfunction.

CTR-CVT	CTRCD
Heart Failure - Biomarkers - Echocardiography - Nuclear Imaging - Cardiac MRI	Symptomatic CTRCD - Biomarkers - Echocardiography - Cardiac MRI - Nuclear Imaging
Myocarditis - Biomarkers - Echocardiography - Cardiac MRI Vascular Toxicity - Cardiac CT Cardiac Arrhythmia - Cardiac CT - Echocardiography Pericardial Disease - Cardiac Biomarkers - Echocardiography - Cardiac CT - Cardiac MRI Valvular Heart Disease - Cardiac Biomarkers - Echocardiography - Cardiac CT - Cardiac MRI	Asymptomatic CTRCD - Biomarkers - Echocardiography - Cardiac MRI - Nuclear Imaging

## Abbreviations

CTR-CVT	cancer therapy-related cardiovascular toxicity
CTRCD	Cancer therapy-related cardiac dysfunction
ESC	European Society of Cardiology
VEGF	Vascular endothelial growth factor
HER2	human epidermal growth factor receptor 2
MEK	mitogen-activated protein kinase
TKI	Tyrosine Kinase Inhibitor
BCR ABL	Breakpoint Cluster Region-Abelson
ALK	anaplastic lymphoma kinase
LVEF	left ventricular ejection fraction
RAF	rapidly accelerated fibrosarcoma
ER	oestrogen receptor
PR	progesterone receptor
HR	hormone receptor
SACT	systemic anti-cancer therapy
DNA	Deoxyribonucleic acid
RNA	Ribonucleic acid
MAPK	mitogen-activated protein kinase
AKT	Ak strain transforming
dFdCTP	Gemcitabine Triphosphate
AI	aromatase inhibitor
CT	Chemotherapy
gBRCA	germline BRCA 1/2 mutation
CDK4/6i	Cyclin-Dependent Kinase 4/6 Inhibitors
ICI	immune checkpoint inhibitor
CI	contraindication
ET	endocrine therapy
BM	brain metastases
SRT	stereotactic radiotherapy
PIK3CA	phosphatidylinositol-4,5-bisphosphate 3-kinase catalytic subunit alpha
ESR1	oestrogen receptor 1
PALB2	partner and localiser of BRCA2
PD-L1	programmed-death ligand 1
CTLA4	Cytotoxic T-lymphocyte-associated protein 4
BMI	body mass index
ntproBNP	N-terminal prohormone of brain natriuretic peptide
NT	natriuretic peptides
CRP	C reactive protein
BNP	brain natriuretic peptide
MRI	magnetic resonance imaging
MPO	myeloperoxidase
PIGF	placental growth factor
GDF-15	growth/differentiation factor-15
IGF1	Insulin-like growth factor 1
cMLC-1	Cardiac Myosin Lightchain 1
ST2/IL33-R	suppression of tumorigenicity 2 protein/interleukin-33R
PON-1	Paraoxonase-1
IL6	Interleukin 6
cfDNA	Circulating cell-free DNA
LVOT	left ventricular outflow tract
LV	left ventricle
RV	right ventricle
TAPSE	tricuspid annular plane systolic excursion
IVC	inferior vena cava
VTI	velocity time integral
EROA	effective regurgitant orifice area
PISA	Proximal Isovelocity Surface Area
CT	computed tomography
RVOT	right ventricular outflow tract
PET-CT	Positron emission tomography/Computed Tomography
TOE	transoesophageal echocardiography
MINOCA	myocardial infarction with non-obstructive coronary arteries
FDG	fluorodeoxyglucose
SPECT	single-photon computed emission tomography
MIBG	iobenguane i-131
ATTR	transthyretin
MUGA	multi-gated acquisition scanning
CIED	cardiovascular implantable electronic devices
CML	chronic myeloid leukaemia
ECG	electrocardiogram
cTN	cardiac troponin
Gy	gray
TTE	transthoracic echocardiography
RCT	randomised control trial
HSCT	haematopoietic stem cell transplant
AC	anthracycline
BC	breast cancer
Tz	trastuzumab
Cycph	cyclophosphamide
MA	meta-analysis
CI	confidence interval
BB	beta blocker
MRA	mineralocorticoid receptor antagonist
ACEi	angiotensin-converting enzyme inhibitor
ARB	angiotensin receptor blocker
NH	neurohormonal
SGLT2	Sodium-glucose cotransporter-2 inhibitor
MDT	multidisciplinary team
ARB	angiotensin receptor blocker
GLS	global longitudinal strain
NOAC	novel oral anticoagulation
